# Health Care Contact Days in Older Adults With Metastatic Cancer

**DOI:** 10.1001/jamanetworkopen.2025.47924

**Published:** 2025-12-09

**Authors:** Arjun Gupta, Shelley A. Jazowski, Avirath U. Vaidya, Stacie B. Dusetzina, Ishani Ganguli

**Affiliations:** 1 Division of Hematology, Oncology, and Transplantation, University of Minnesota, Minneapolis; 2 Department of Social Sciences and Health Policy, Wake Forest University School of Medicine, Winston-Salem, North Carolina; 3 Department of Health Policy, Vanderbilt University School of Medicine, Nashville, Tennessee; 4 Vanderbilt-Ingram Cancer Center, Nashville, Tennessee; 5 Division of General Internal Medicine and Primary Care, Brigham and Women’s Hospital, Harvard Medical School, Boston, Massachusetts

## Abstract

**Importance:**

Older adults with metastatic solid cancers experience substantial treatment burdens. Advances in cancer therapeutics and evolutions in care over the years have changed the experience of cancer diagnosis and treatment, but it is unknown how health care contact days have evolved over time.

**Objective:**

To assess the burden of and trends in health care contact days among older adults diagnosed with 4 common metastatic cancers.

**Design, Setting, and Participants:**

This cohort study used the Surveillance, Epidemiology, and End Results–Medicare linked database to identify traditional Medicare beneficiaries 66 years and older who were diagnosed with metastatic breast, colorectal, lung, and prostate cancers from January 2008 to February 2019. Analyses were conducted from February 2024 to January 2025.

**Exposures:**

Cancer type and year of diagnosis.

**Main Outcomes and Measures:**

Health care contact days (sum of ambulatory [days with a clinician visit, test, imaging, procedure, or treatment] and institutional [days in a hospital, emergency department, skilled nursing facility, or inpatient hospice] days) in the year after diagnosis were assessed. Changes in contact days over time were examined using multivariable negative binomial regression models.

**Results:**

A total of 55 806 beneficiaries (14 827 [26.6%] diagnosed at 71-75 years of age; 29 347 [52.6%] male) with metastatic cancer (6495 breast, 10 232 colorectal, 27 340 lung, and 11 739 prostate) who survived 1 year or more after diagnosis were studied. In the year after diagnosis, beneficiaries with colorectal cancer had the highest mean (SD) contact days (62.9 [48.1]), followed by those with lung (60.2 [47.0]), breast (48.7 [47.9]), and prostate (40.1 [42.4]) cancers. Across all cancer types, mean contact days increased from 2008 to 2019 with a prominent increase in ambulatory days from 2016 onward; the largest increase in contact days was observed for breast cancer (44.9 [95% CI, 38.7-52.2] to 57.6 [95% CI, 46.9-70.8]).

**Conclusions and Relevance:**

This cohort study of older traditional Medicare beneficiaries who were diagnosed with common metastatic cancers found a mean of 40.1 to 62.9 health care contact days in the year after diagnosis. Health care contact days increased from 2008 to 2019, suggesting that treatment advancements and/or care inefficiencies may have imposed additional burdens on beneficiaries.

## Key Points

**Question:**

What are the patterns and changes in health care contact days among older adult Medicare beneficiaries with common metastatic cancers?

**Findings:**

In this cohort study of 55 806 Medicare beneficiaries with metastatic breast, colorectal, lung, and prostate cancers who survived 1 year or more, participants experienced a mean of 40.1 to 62.9 contact days in the year after diagnosis. Across all cancer types, contact days increased from 2008 to 2019, with a large increase in ambulatory days from 2016 onward.

**Meaning:**

This study’s results suggest that oncology teams should discuss expected contact days and related burdens when developing care goals and treatment plans with older adult patients.

## Teaser

This cohort study examines patterns and changes in health care contact days over time among traditional Medicare beneficiaries diagnosed with metastatic breast, colorectal, lung, and prostate cancers.

## Introduction

Health care contact days, defined as days with health care interaction outside the home, serve as a patient-centered measure of how much of life is consumed receiving health care.^1,2,3^ Prior work has demonstrated that such days are particularly frequent for patients with metastatic cancer, who spend approximately 1 in 5 days interacting with the health care system.^4,5,6,7,8,9^ Although many of these days represent necessary and goal-aligned care, most patients would want to achieve their health goals with the minimum necessary contact days because these days can also impose logistical, financial, and/or care management burdens.^10,11^ Given the life-limiting nature of metastatic cancer and the intensive treatments associated with care, documenting the typical frequency and composition of health care contact after diagnosis can help set expectations for and inform patients’ care decisions. Health systems can use such information to ensure appropriate support for patients and families, and policymakers can use it to identify levers to optimize contact days.

Health care contact days are particularly relevant to older adults because they often face greater burdens related to cancer, its treatment, and comorbidities, as well as worse oncologic outcomes.^12,13,14^ In 2025, more than 1 million Americans are projected to be diagnosed with 1 of 4 of the highest-incidence cancers—breast, colorectal, lung, and prostate—which account for half of new cancer diagnoses in the US,^15^ and most new cancer diagnoses and deaths continue to occur in older adults.^15,16^ Despite screening and early detection efforts, a substantial number of cancers are metastatic at diagnosis.^17^ At the same time, advances in cancer therapeutics, such as an expanding array of treatments, and evolutions in care, such as increasingly complex and subspecialized care, have changed the experience of diagnosis and treatment for older adults with metastatic cancer. Our objectives were to examine the patterns of and changes in health care contact days over time among traditional Medicare beneficiaries diagnosed with metastatic breast, colorectal, lung, and prostate cancers.

## Methods

### Data Source and Study Population

We used the Surveillance, Epidemiology, and End Results (SEER)–Medicare linked database to identify older adults in traditional Medicare who were diagnosed with metastatic breast, colorectal, lung, and prostate cancer from January 1, 2008, to February 28, 2019 (eFigures 1-4 in Supplement 1). Eligible beneficiaries included those who were 66 years or older at diagnosis, continuously enrolled in traditional Medicare Parts A and B in the year before and after diagnosis, and not originally eligible for benefits due to end-stage renal disease. The Vanderbilt University Medical Center institutional review board approved this cohort study and waived informed consent because the data analyzed were deidentified. This study followed the Strengthening the Reporting of Observational Studies in Epidemiology (STROBE) reporting guideline.

### Outcome

The primary outcome was health care contact days in the year after diagnosis, defined as days interacting with the health care system outside the home.^1,2,18,19,20^ Using previously described methods,^1^ we identified days spent in an institutional setting: hospital, emergency department (ED),^21^ skilled nursing facility, or inpatient hospice (eTable 1 in Supplement 1). We used the 2023 Restructured Berenson-Eggers Type of Service (BETOS) Classification System taxonomy to identify days receiving care in the ambulatory setting, including clinician visits, tests (eg, blood tests), imaging (eg, radiographs), procedures (eg, breast or gastrointestinal procedures), or treatments (eg, chemotherapy). Consistent with prior research,^1,18,19^ we applied the following hierarchy when identifying specific contact days: (1) hospital, (2) ED, (3) skilled nursing facility, (4) inpatient hospice, and (5) any ambulatory care (eg, if, on a given day, a beneficiary was admitted to the hospital from the ED, then it was only counted as a hospital day).

### Covariates

Covariates included the following sociodemographic and health-related characteristics: age at diagnosis, sex, Research Triangle Institute race and ethnicity (Black or African American, Hispanic, non-Hispanic White, and other [Asian or Pacific Islander, Alaska Native or American Indian, other, or unknown]) because race and ethnicity can be associated with care access and experiences,^22^ low income (defined as receipt of full or partial Medicare Part D low-income subsidies), urbanicity (measured with rural-urban continuum codes known as Beale codes), census region (based on location of SEER registry), year of diagnosis, comorbidities (measured in the year before and month of diagnosis using the Klabunde modification of the Charlson score),^23^ and receipt of chemotherapy and/or radiation therapy within 180 days after diagnosis (measured with the 2023 Restructured BETOS Classification System taxonomy).

### Statistical Analysis

For each cancer type, we used descriptive statistics to describe total health care contact days and their components across the study period (2008-2020) and stratified by year of diagnosis (2008-2019). We used negative binomial regression to assess the association of sociodemographic and health-related characteristics with total health care contact days across the study period, then to estimate total contact days stratified by year of diagnosis with adjustment for these characteristics to understand how contact days changed over time. Analyses were conducted from February 2024 to January 2025 using SAS Studio, version 9.4 (SAS Institute Inc). Statistical tests were 2-sided, and *P* < .05 denoted statistical significance.

### Sensitivity Analysis

We conducted a sensitivity analysis including decedents and beneficiaries switching from traditional Medicare.^24^ Specifically, we identified contact days until Medicare disenrollment, death, or 1 year after diagnosis (whichever came first) among beneficiaries who were continuously enrolled in traditional Medicare Parts A and B in the year before and at least 1 month after diagnosis.

## Results

### Cohort Characteristics

Among the 55 806 traditional Medicare beneficiaries studied (14 827 [26.6%] diagnosed at 71-75 years of age, 29 347 [52.6%] male and 26 459 [47.4%] female), 27 340 (49.0%), 11 739 (20.4%), 10 232 (18.3%), and 6495 (11.6%) were diagnosed with metastatic lung, prostate, colorectal, and breast cancer, respectively (Table 1). Across the 4 cancer types, most beneficiaries were non-Hispanic White (lung: 22 166 [81.1%]; prostate: 8928 [76.1%]; colorectal: 7963 [77.8%]; breast: 5227 [80.5%]), resided in big metropolitan areas (lung: 15 494 [56.7%]; prostate: 6433 [54.8%]; colorectal: 5882 [57.5%]; breast: 3770 [58.0%]), and were treated with chemotherapy and/or radiation therapy in the 180 days after diagnosis (lung: 18 939 [69.3%]; prostate: 8857 [75.5%]; colorectal: 6722 [65.7%]; breast: 3396 [52.6%]).

**Table 1.** Baseline Sociodemographic and Health-Related Characteristics by Cancer Type

Characteristic	No. (%) of participants			
	Breast cancer (n = 6495)	Colorectal cancer (n = 10 232)	Lung cancer (n = 27 340)	Prostate cancer (n = 11 739)
Age group, y				
≤70	1915 (29.5)	3219 (31.5)	8519 (31.2)	2699 (23.0)
71-75	1663 (25.6)	2747 (26.9)	7723 (28.3)	2694 (23.0)
76-80	1233 (19.0)	2040 (19.9)	5788 (21.2)	2441 (20.8)
≥81	1684 (25.9)	2226 (21.8)	5310 (19.4)	3905 (33.3)
Sex				
Male	99 (1.5)	5058 (49.4)	12 451 (45.5)	11 739 (100.0)
Female	6396 (98.5)	5174 (50.6)	14 889 (54.5)	0
Race and ethnicity				
Black or African American	630 (9.7)	913 (8.9)	2059 (7.5)	1298 (11.1)
Hispanic	379 (5.8)	736 (7.2)	1270 (4.7)	891 (7.6)
White	5227 (80.5)	7963 (77.8)	22 166 (81.1)	8928 (76.1)
Other or unknown^a^	259 (4.0)	620 (6.1)	1845 (6.8)	622 (5.3)
Low-income subsidy^b^				
Full or partial	1321 (20.3)	1961 (19.2)	5208 (19.1)	1884 (16.1)
None	5174 (79.7)	8271 (80.8)	22 132 (81.0)	9855 (84.0)
Urbanicity^c^				
Big metropolitan	3770 (58.0)	5882 (57.5)	15 494 (56.7)	6433 (54.8)
Metropolitan	1779 (27.4)	2723 (26.6)	7487 (27.4)	3432 (29.2)
Urban	355 (5.5)	574 (5.6)	1551 (5.7)	687 (5.9)
Less urban	499 (7.7)	871 (8.5)	2321 (8.5)	986 (8.4)
Rural	92 (1.4)	182 (1.8)	485 (1.8)	201 (1.7)
Region^d^				
Northeast	2311 (35.6)	3633 (35.5)	9335 (34.1)	3862 (32.9)
Midwest	480 (7.4)	762 (7.5)	2208 (8.1)	880 (7.5)
South	2075 (32.0)	3152 (30.8)	8521 (31.2)	3240 (27.6)
West	1629 (25.1)	2685 (26.2)	7276 (26.6)	3757 (32.0)
No. of comorbidities^e^				
0	3659 (56.3)	4984 (48.7)	9975 (36.5)	6103 (52.0)
1	1347 (20.7)	2305 (22.5)	7234 (26.5)	2204 (18.8)
≥2	1489 (22.9)	2943 (28.8)	10 131 (37.1)	3432 (29.2)
Treated in 180 d after diagnosis^f^				
Yes	3396 (52.3)	6722 (65.7)	18 939 (69.3)	8857 (75.5)
No	3099 (47.7)	3510 (34.3)	8401 (30.7)	2882 (24.6)
Year of diagnosis^g^				
2008	564 (8.7)	1038 (10.1)	2487 (9.1)	777 (6.6)
2009	563 (8.7)	979 (9.6)	2414 (8.8)	788 (6.7)
2010	563 (8.7)	1035 (10.1)	2421 (8.9)	835 (7.1)
2011	570 (8.8)	930 (9.1)	2592 (9.5)	866 (7.4)
2012	574 (8.8)	901 (8.8)	2437 (8.9)	891 (7.6)
2013	582 (9.0)	919 (9.0)	2446 (9.0)	992 (8.5)
2014	592 (9.1)	911 (8.9)	2468 (9.0)	1112 (9.5)
2015	607 (9.5)	909 (8.9)	2562 (9.4)	1216 (10.4)
2016	614 (9.4)	806 (7.9)	2340 (8.6)	1306 (11.1)
2017	560 (8.6)	776 (7.6)	2378 (8.7)	1359 (11.6)
2018	611 (9.4)	892 (8.7)	2364 (8.7)	1353 (11.5)
2019	95 (1.5)	136 (1.3)	431 (1.6)	244 (2.1)

^a^ Other and unknown race and ethnicity included Asian or Pacific Islander, Alaska Native or American Indian, other, or unknown.

^b^ Receipt of Medicare Part D low-income subsidies at diagnosis was an indicator of low income.

^c^ Urbanicity was defined using rural-urban continuum codes known as Beale codes. Beneficiaries missing urbanicity were categorized as Big Metropolitan (n = 2 beneficiaries with lung cancer; n = 1 beneficiary with prostate cancer).

^d^ Surveillance, Epidemiology, and End Results registries were categorized into US Census regions.

^e^ Comorbidities were measured in the year before and the month of diagnosis using the Klabunde modification of the Charlson score.

^f^ Treatment with radiation therapy and/or chemotherapy was measured in the 180 days after diagnosis with restructured Berenson-Eggers Type of Service (BETOS) Classification System taxonomy. The BETOS code for chemotherapy includes treatment with immunotherapy.

^g^ Cohort included beneficiaries diagnosed from January 1, 2008, to February 28, 2019.

### Composition of Health Care Contact Days

In the year after diagnosis, beneficiaries with colorectal cancer had the highest mean (SD) health care contact days (62.9 [48.1]) followed by those with lung (60.2 [47.0]), breast (48.7 [47.9]), and prostate cancers (40.1 [42.4]) (Table 2). Ambulatory days comprised most contact days and accounted for a mean (SD) of 29.5 (28.9) postdiagnosis days for prostate cancer and 46.7 (35.0) postdiagnosis days for lung cancer. When examining individual components of health care contact days, we found the highest mean (SD) days for treatments (lung: 26.1 [24.1]; colorectal: 23.6 [22.0]; breast: 20.2 [22.5]; prostate: 15.5 [19.6]), clinician visits (lung: 23.2 [15.4]; colorectal: 23.1 [16.1]; breast: 18.5 [13.5]; prostate: 16.3 [11.9]) and tests (lung: 21.0 [15.2]; colorectal: 22.2 [15.9]; breast: 16.8 [13.7]; prostate: 13.8 [11.2]).

**Table 2.** Health Care Contact Days and Their Composition by Cancer Type^a^

Variable	Health care contact days, mean (SD)			
	Breast cancer (n = 6495)	Colorectal cancer (n = 10 232)	Lung cancer (n = 27 340)	Prostate cancer (n = 11 739)
Total contact days^b^	48.7 (47.9)	62.9 (48.1)	60.2 (47.0)	40.1 (42.4)
Total institutional days^c^	13.5 (34.9)	18.5 (34.9)	13.5 (30.7)	10.5 (28.7)
Total ambulatory days^d^	35.2 (32.8)	44.4 (32.7)	46.7 (35.0)	29.5 (28.9)
Hospital or inpatient	4.8 (11.4)	9.5 (14.0)	6.6 (11.8)	4.1 (10.5)
Emergency department	0.8 (1.9)	1.0 (2.1)	1.1 (2.4)	1.0 (2.3)
Skilled nursing facility	7.1 (26.1)	7.0 (23.9)	4.5 (19.5)	5.1 (21.1)
Inpatient hospice	0.8 (14.0)	1.1 (15.4)	1.3 (16.1)	0.4 (8.6)
Visits	18.5 (13.5)	23.1 (16.1)	23.2 (15.4)	16.3 (11.9)
Tests	16.8 (13.7)	22.2 (15.9)	21.0 (15.2)	13.8 (11.2)
Imaging	5.7 (4.6)	5.4 (3.9)	7.5 (5.0)	4.4 (3.9)
Procedures	3.4 (6.3)	4.7 (6.9)	4.1 (5.7)	3.0 (4.5)
Treatment	20.2 (22.5)	23.6 (22.0)	26.1 (24.1)	15.5 (19.6)

^a^ Health care contact days were measured in the year after diagnosis. When identifying specific types of contact days, the following hierarchy was applied: (1) hospital or inpatient, (2) emergency department, (3) skilled nursing facility, (4) inpatient hospice, and (5) any ambulatory care (eg, if beneficiary was admitted to the hospital from the emergency department on a given day, it was only counted as an inpatient or hospital day).

^b^ Total contact days are the sum of institutional and ambulatory days.

^c^ Total institutional days are the sum of hospital or inpatient, emergency department, skilled nursing facility, and hospice days.

^d^ Total ambulatory days are the sum of visits, tests, imaging, procedures, and treatment days.

### Health Care Contact Days Over Time

Across the 4 cancer types, health care contact days increased from 2008 to 2019, with the largest increase observed for breast cancer (44.9 [95% CI, 38.7-52.2] to 57.6 [95% CI, 46.9-70.8]) (Figure; eTable 4 in Supplement 1). Although mean institutional days remained relatively consistent over time, mean (SD) ambulatory days increased from 2016 to 2019 for each cancer type (breast: 35.8 [33.7] to 46.2 [31.8]; colorectal: 45.5 [31.0] to 55.6 [27.0]; lung: 46.2 [32.1] to 52.4 [32.1]; prostate: 31.0 [28.8] to 35.7 [26.3]).

**Figure.**
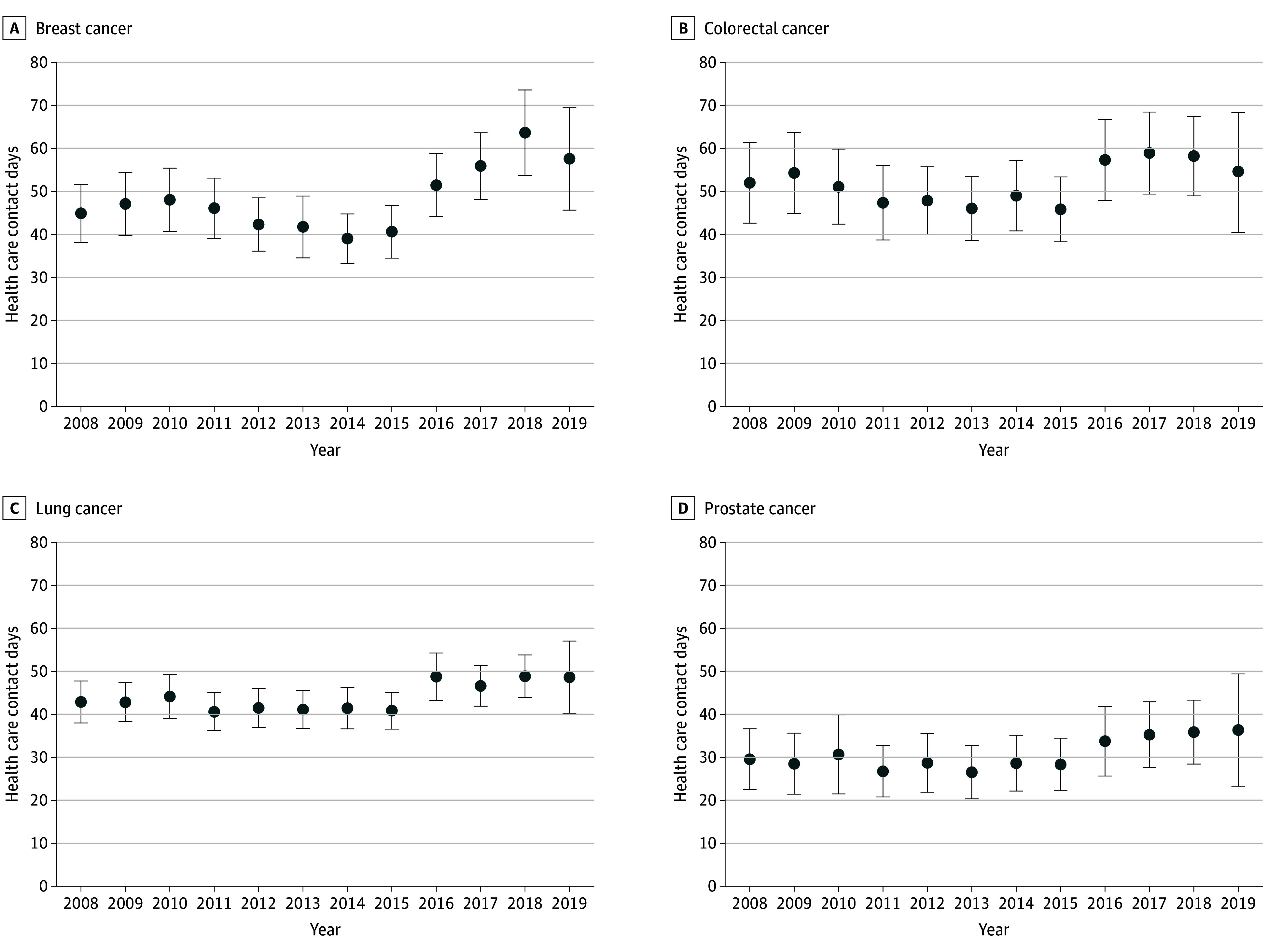
Mean Health Care Contact Days by Year of Diagnosis and Cancer Type Figure displays the adjusted mean contact days by year of diagnosis for beneficiaries who survived 1 year or more after diagnosis. For each cancer type, adjusted means were estimated with a negative binomial regression model that controlled for sociodemographic and health-related characteristics. Error bars indicate 95% CIs.

### Factors Associated With Health Care Contact Days

Across the 4 cancer types, the sociodemographic and health-related characteristics associated with more contact days were non-Hispanic White and Black or African American race and ethnicity, residing in census regions other than the South, having low income, receipt of chemotherapy and/or radiation therapy in the 180 days after diagnosis, and having one or more comorbidities (eTable 6 in Supplement 1).

### Sensitivity Analysis

When expanding the cohort to those who died or disenrolled from traditional Medicare Parts A and B within the year of diagnosis, we observed 164 747 beneficiaries (eFigures 1-4 and eTable 2 in Supplement 1). Findings were similar to our primary results; however, health care contact days represented a larger share of their post-diagnosis days in this cohort (39.0 of 229.5 days alive and enrolled in 2008 to 36.5 of 121.7 days in 2019 vs 40.1 of 365 days in 2008 to 62.9 of 365 days in the primary analysis) (eTables 3, 5, and 7 in Supplement 1). From 2008 to 2019, 1-year survival increased for beneficiaries with lung (2487 of 11 322 to 431 of 1412) and prostate (777 of 1230 to 244 of 342) cancers, but we observed a slight decrease in 1-year survival for those with breast cancer (564 of 972 to 95 of 175) (eFigure 5 in Supplement 1).

## Discussion

In this population-based study, traditional Medicare beneficiaries who were 66 years or older with metastatic breast, colorectal, lung, and prostate cancers experienced a mean of 40.1 to 62.9 health care contact days in the year after diagnosis. The most common source of contact days was ambulatory care, specifically administration of treatments, receipt of tests, and visits with clinicians. Across the 4 cancer types, health care contact days increased from 2008 to 2019, with a prominent increase in ambulatory days from 2016 onward.

This study is the first, to our knowledge, to examine the frequency of and changes in health care contact days among older traditional Medicare beneficiaries with 4 common metastatic cancers. Prior studies^4,5,7,20,25,26^ assessing rates of contact days among adults with metastatic cancer have been limited to single institutions or focus solely on trial participants or patients with one cancer. The current study captured a time of intense clinical trial investigations, drug approvals, and treatment uptake in the US and was well-suited to examine parallel changes in contact days. Increasing contact days observed during the study period should prompt oncology care teams to recognize the time burden associated with metastatic cancer care and consider expected health care contact days when discussing care goals and choosing treatment plans with patients.

In our primary analysis, we found that older traditional Medicare beneficiaries with 4 common metastatic cancers who survived 1 year or longer spent 11.0% to 17.2% of the first year after diagnosis receiving health care outside the home, whereas all such patients (regardless of survival time) spent 17.0% to 30.0% of their remaining days interacting with the health care system. Our findings build on prior estimates of 20% to 30% observed in studies focused on single cancers, single institutions, trial participants, and those receiving systemic therapies.^4,5,7,20,25,26^ Notably, the primary cohort in this study (surviving ≥1 year) was just one-third the size of the sensitivity analysis cohort, which not only highlights the incredible early mortality faced by these beneficiaries but also indicates that the results of the primary analysis may be optimistic for the average older adult with incident metastatic cancer. The higher frequency of contact days among beneficiaries with shorter survival is critically relevant when making decisions regarding multiple lines of cancer-directed treatment because each line of treatment typically offers diminishing returns in terms of survival benefit but higher rates of contact days.^20^

Across the 4 cancer types, we observed an increase in total contact days and ambulatory days from 2008 to 2019. This finding may partially be explained by the greater number of treatment options that have become available during the study period—the US Food and Drug Administration approved 332 anticancer therapies from 2009 to 2020.^27^ Although most of these drug approvals were in the later-line setting, the first-line standard of care has significantly changed for some cancers. For example, in 2008, the standard treatment for newly diagnosed metastatic prostate cancer was solely androgen deprivation therapy, typically delivered as an injection every 3 months; more recently, the standard has become androgen deprivation therapy in combination with intravenous chemotherapy and/or other hormonal agents.^28,29^ These treatments are associated with monitoring needs, such as frequent bloodwork, and short- and long-term multiorgan toxic effects that may require specialist visits.^30,31^ The observed increase in contact days with treatments, tests, and clinician visits suggests that the adoption of novel treatments may have contributed to increasing total contact and ambulatory days over time, which is particularly concerning because 1-year survival rates improved minimally for beneficiaries with lung and prostate cancers and even decreased for those with breast cancer.

Another potential explanation for increasing ambulatory and total contact days over time is that ambulatory care is often fragmented and uncoordinated,^10,19,32^ maybe more so in recent years as cancer care becomes more complex and subspecialized. Prior research on health care contact days has demonstrated missed opportunities to coordinate ambulatory care, for example, by scheduling visits and other services (eg, tests and imaging) on the same day.^1,5,10,18,19,33^ This is particularly relevant for older adults with metastatic cancer who may require care for comorbidities in addition to cancer care. Future research should build on our findings and evaluate the role of specific treatments, changes in treatments over time, and care fragmentation and coordination on health care contact days.

Consistent with prior research, we observed that health care contact days varied by sociodemographic and health-related characteristics.^1,7,18,19,20^ Specifically, Hispanic beneficiaries and those residing in the South experienced fewer contact days, which could reflect structural barriers (eg, lack of reliable transportation), geographic barriers (eg, medical deserts and longer travel distances), and/or financial barriers (eg, high cost-sharing) to care.^19,34,35,36,37,38^ Similar to previous research on patients with cancer,^19^ we found that low-income beneficiaries experienced more health care contact days, which could be due to worse health status (incompletely adjusted for in the multivariable model) and the need for more care. Indeed, this study and prior work^1,19^ found a dose-dependent association between health care contact days and the number of comorbidities. Future research should focus on understanding how to optimize contact days and addressing known barriers to ensure quality of and equal access to care.

### Limitations

Our study had limitations. First, our analysis focused on older traditional Medicare beneficiaries diagnosed with incident metastatic cancer; thus, findings may not generalize to those who are younger than 66 years, covered by private or other public insurance, diagnosed with early-stage or recurrent cancer, or living outside the US. Second, although our models accounted for receipt of chemotherapy and/or radiation therapy in the 180 days after diagnosis, we did not identify the use of other systemic therapies (eg, self-administered drugs), surgery, or palliative or supportive care services. Third, we were unable to discern the reasons for not initiating chemotherapy or radiation therapy (eg, beneficiary was ineligible for or declined treatment). Fourth, we did not assess the appropriateness, quality, or efficiency of health care contact days (eg, receipt of guideline-concordant care, coordination of health care encounters).

## Conclusions

In this cohort study of older traditional Medicare beneficiaries who were diagnosed with 4 common metastatic cancers, we observed a mean of 40.1 to 62.9 health care contact days in the year after diagnosis (accounting for 11.0% to 17.2% of the year). Across the 4 cancer types, contact days increased from 2008 to 2019, suggesting that treatment advancements and/or inefficiencies in care may have imposed additional burdens on beneficiaries. Understanding the drivers of health care contact days and beneficiaries’ preferences for care are critical to reduce the time burden associated with cancer care and ensure shared decision-making when determining the appropriate care for older adults with metastatic cancer.

## Notes

Supplement 1. **eFigure 1.** Study Flow Diagram for Breast Cancer Cohort **eFigure 2.** Study Flow Diagram for Colorectal Cancer Cohort **eFigure 3.** Study Flow Diagram for Lung Cancer Cohort **eFigure 4.** Study Flow Diagram for Prostate Cancer Cohort **eTable 1. **Health Care Contact Days Definitions and Identification **eTable 2.** Baseline Sociodemographic and Health-related Characteristics by Cancer Type (Secondary Cohort) **eTable 3.** Health Care Contact Days By Cancer Type (Secondary Cohort) **eTable 4.** Health Care Contact Days by Year of Diagnosis and Cancer Type (Primary Cohort) **eTable 5.** Health Care Contact Days by Year of Diagnosis and Cancer Type (Secondary Cohort) **eTable 6.** Association of Sociodemographic and Health-related Characteristics with Health Care Contact Days (Primary Cohort) **eTable 7.** Association of Sociodemographic and Health-related Characteristics with Health Care Contact Days (Secondary Cohort) **eFigure 5**. 12-Month Survival by Year of Diagnosis and Cancer Type (Secondary Cohort)

Supplement 2.
Data Sharing Statement
