# Supplementary material for: Health Care Contact Days in Older Adults With Metastatic Cancer
Source: JAMA Netw Open. 2025 Dec 9;8(12):e2547924. doi: 10.1001/jamanetworkopen.2025.47924 (PMC12690425; doi:10.1001/jamanetworkopen.2025.47924)
Supplement: Supplement 1. — eFigure 1. Study Flow Diagram for Breast Cancer Cohort eFigure 2. Study Flow Diagram for Colorectal Cancer Cohort eFigure 3. Study Flow Diagram for Lung Cancer Cohort eFigure 4. Study Flow Diagram for Prostate Cancer Cohort eTable 1. Health Care Contact Days Definitions and Identification eTable 2. Baseline Sociodemographic and Health-related Characteristics by Cancer Type (Secondary Cohort) eTable 3. Health Care Contact Days By Cancer Type (Secondary Cohort) eTable 4. Health Care Contact Days by Year of Diagnosis and Cancer Type (Primary Cohort) eTable 5. Health Care Contact Days by Year of Diagnosis and Cancer Type (Secondary Cohort) eTable 6. Association of Sociodemographic and Health-related Characteristics with Health Care Contact Days (Primary Cohort) eTable 7. Association of Sociodemographic and Health-related Characteristics with Health Care Contact Days (Secondary Cohort) eFigure 5. 12-Month Survival by Year of Diagnosis and Cancer Type (Secondary Cohort) [file jamanetwopen-e2547924-s001.pdf]

Supplemental Online Content

Gupta A, Jazowski SA, Vaidya AU, Dusetzina SB, Ganguli I. Health care contact days in older adults with metastatic cancer. *JAMA Netw Open*. 2025;8(12):e2547924. doi:10.1001/jamanetworkopen.2025.47924

eFigure 1. Study Flow Diagram for Breast Cancer Cohort
eFigure 2. Study Flow Diagram for Colorectal Cancer Cohort
eFigure 3. Study Flow Diagram for Lung Cancer Cohort
eFigure 4. Study Flow Diagram for Prostate Cancer Cohort
eTable 1. Health Care Contact Days Definitions and Identification
eTable 2. Baseline Sociodemographic and Health-related Characteristics by Cancer Type (Secondary Cohort)
eTable 3. Health Care Contact Days By Cancer Type (Secondary Cohort)
eTable 4. Health Care Contact Days by Year of Diagnosis and Cancer Type (Primary Cohort)
eTable 5. Health Care Contact Days by Year of Diagnosis and Cancer Type (Secondary Cohort)
eTable 6. Association of Sociodemographic and Health-related Characteristics with Health Care Contact Days (Primary Cohort)
eTable 7. Association of Sociodemographic and Health-related Characteristics with Health Care Contact Days (Secondary Cohort)
eFigure 5. 12-Month Survival by Year of Diagnosis and Cancer Type (Secondary Cohort)

This supplemental material has been provided by the authors to give readers additional information about their work.

© 2025 Gupta A et al. *JAMA Network Open*.

eFigure 1. Study Flow Diagram for Breast Cancer Cohort

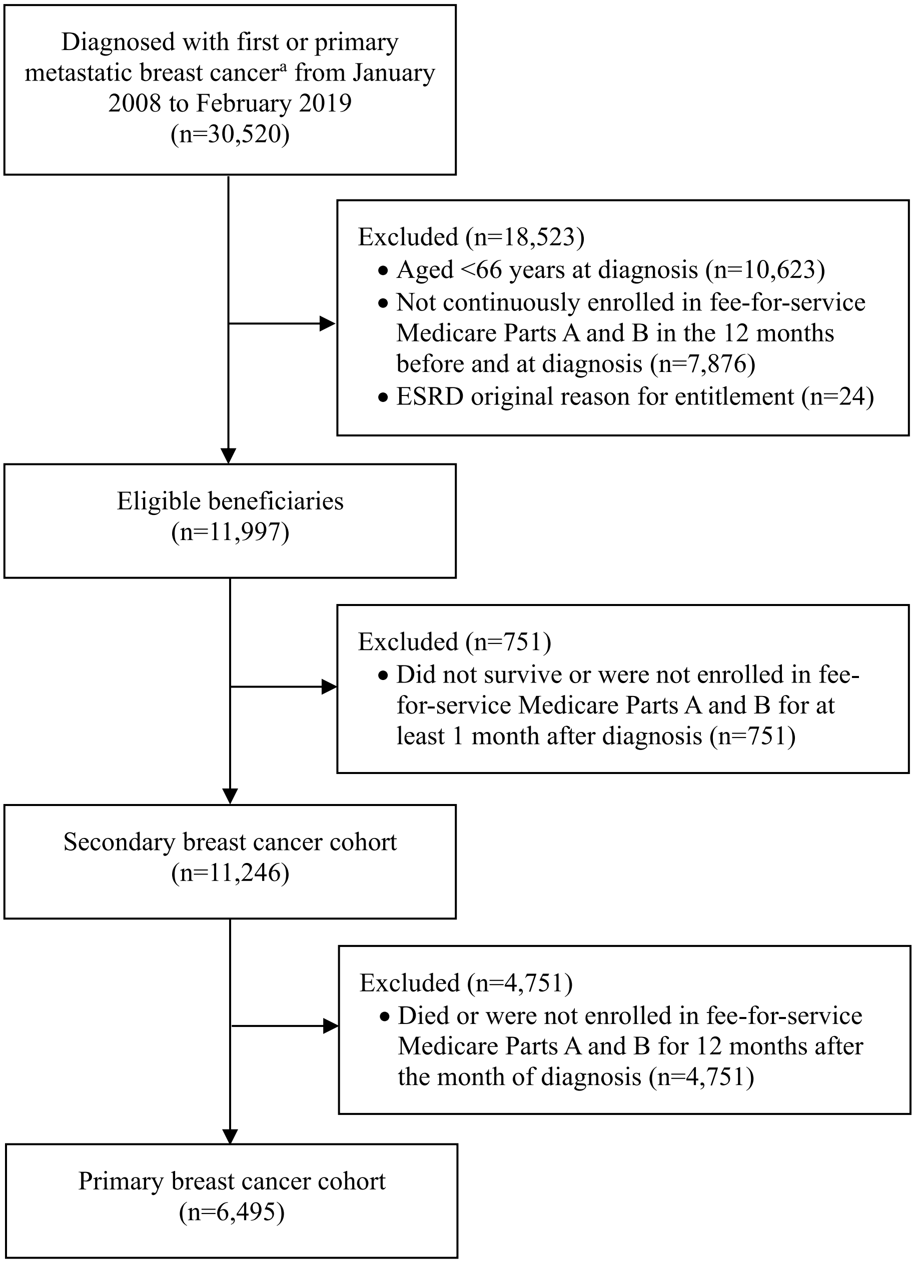

Abbreviations: ESRD, end-stage renal disease
[a] Distant summary stage (cancer has spread to other parts of the body) was used to identify beneficiaries diagnosed with metastatic disease.

© 2025 Gupta A et al. *JAMA Network Open*.

eFigure 2. Study Flow Diagram for Colorectal Cancer Cohort

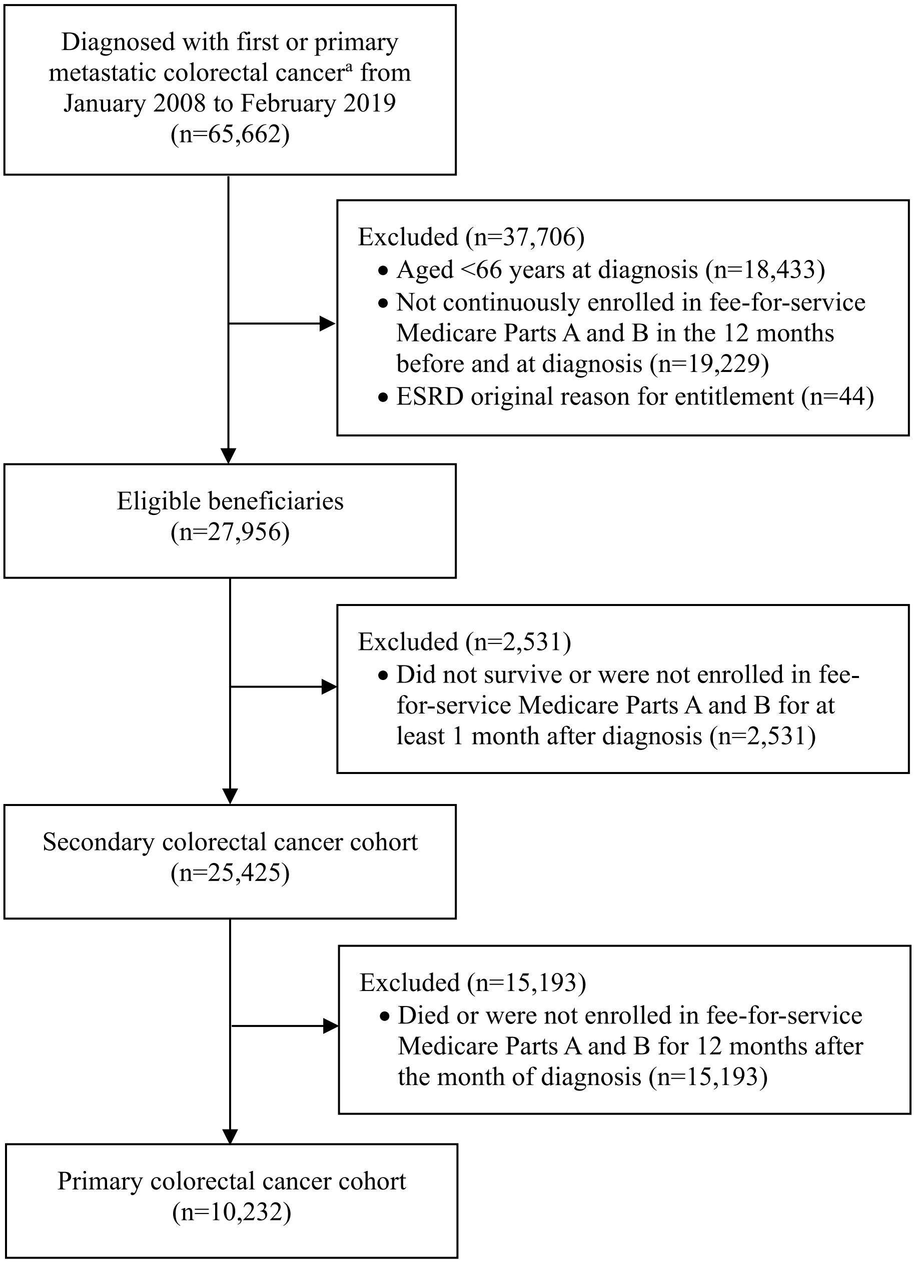

Abbreviations: ESRD, end-stage renal disease
[a] Distant summary stage (cancer has spread to other parts of the body) was used to identify beneficiaries diagnosed with metastatic disease.

© 2025 Gupta A et al. *JAMA Network Open*.

eFigure 3. Study Flow Diagram for Lung Cancer Cohort

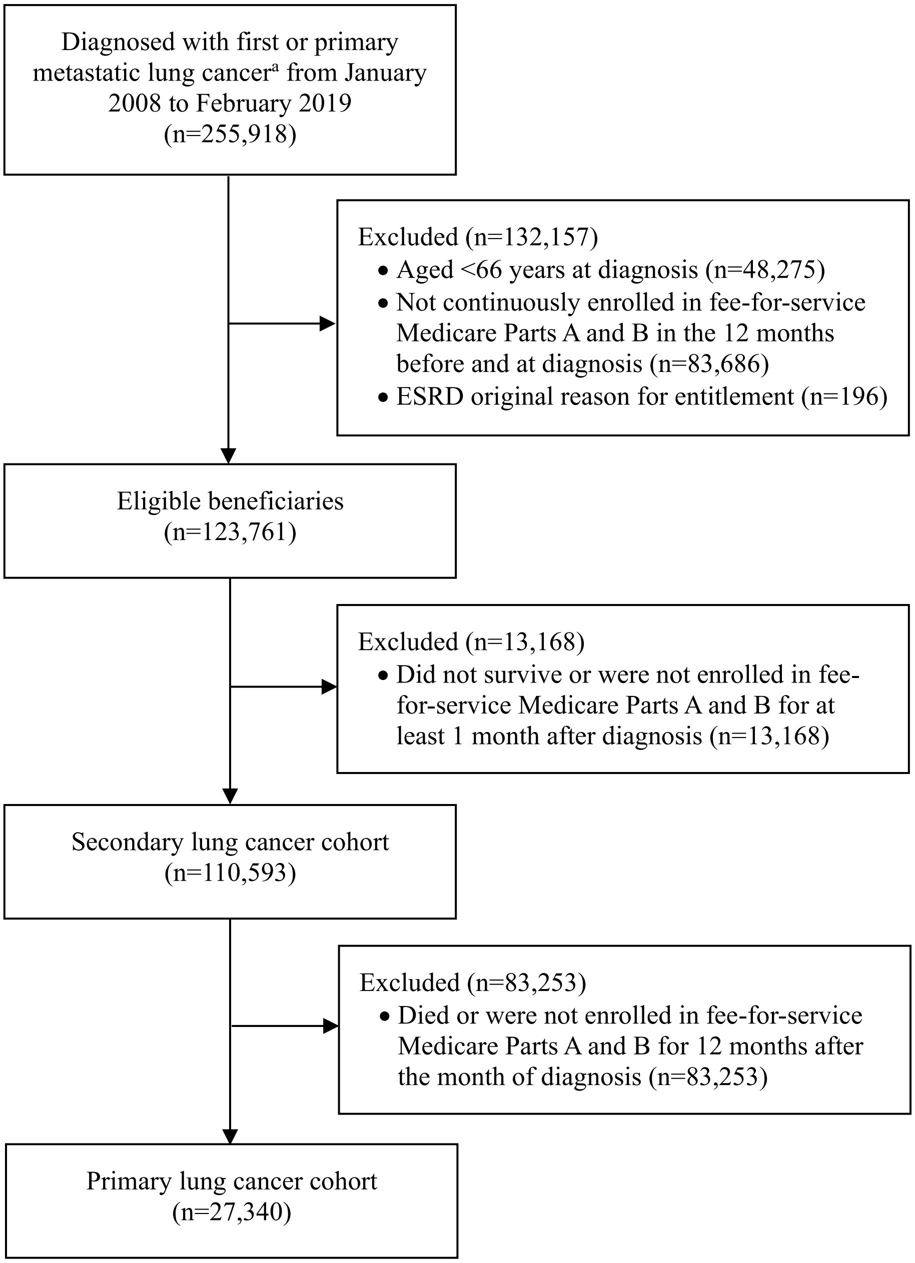

Abbreviations: ESRD, end-stage renal disease
[a] Distant summary stage (cancer has spread to other parts of the body) was used to identify beneficiaries diagnosed with metastatic disease.

© 2025 Gupta A et al. *JAMA Network Open*.

eFigure 4. Study Flow Diagram for Prostate Cancer Cohort

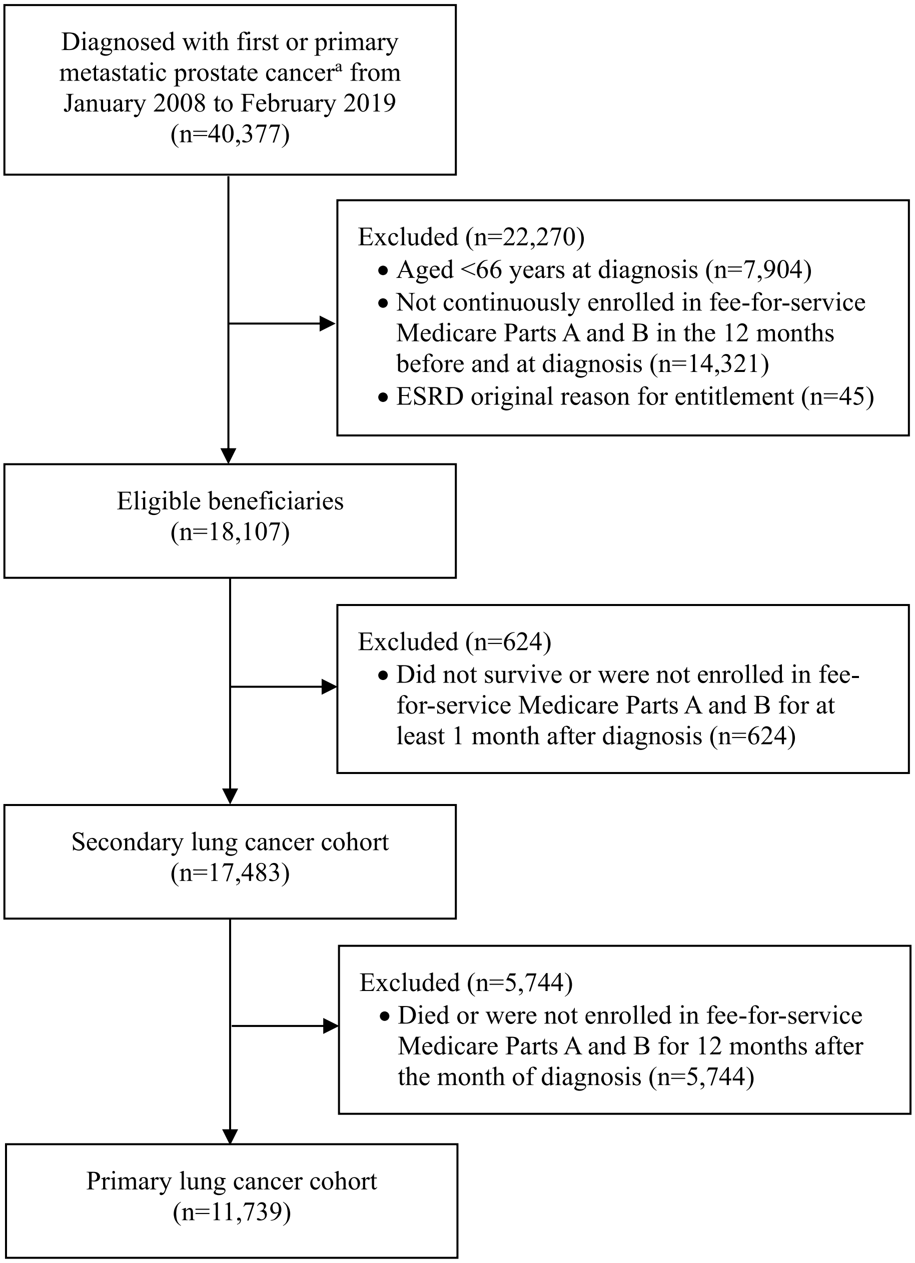

Abbreviations: ESRD, end-stage renal disease
[a] Distant summary stage (cancer has spread to other parts of the body) was used to identify beneficiaries diagnosed with metastatic disease.

© 2025 Gupta A et al. *JAMA Network Open*.

eTable 1. Health Care Contact Days Definitions and Identification

Variable	Variable Names	Definition	Date(s) definition	File
Inpatient	NCH_CLM_TYPE_CD<br>SS_LS_SNF_IND_CD	Days in which beneficiary is hospitalized in the inpatient setting<br>SS_LS_SNF_IND_CD not equal to N<br>NCH_CLM_TYPE_CD not equal to 20	ADMSN_DT to DSCHRG_DT	MedPAR
Emergency department visit	HCPCS;<br>REV_CNTR	Outpatient: HCPCS: any of 99281-99285 or 99291 **AND** REV_CNTR = 0450-0452,0456, 0459, 0981) OR ED Observation (defined as HCPCS G0378 or G0379 **AND** REV_CNTR = 0760, 0762<br>NCH: HCPCS_CD = 99281-5 or 99291 **AND** LINE_PLACE_OF_SRVC_CD = 23.	CLM_FROM_DT to CLM_THRU_DT	Outpatient, NCH
Skilled nursing facility	NCH_CLM_TYPE_CD<br>SS_LS_SNF_IND_CD	Days in which beneficiary is in a skilled nursing facility<br>NCH_CLM_TYPE_CD = 20 or 30<br>SS_LS_SNF_IND_CD = N or missing	ADMSN_DT to DSCHRG_DT If DSCHRG_DT was missing then date was calculated based on length of stay	MedPAR
Hospice	CLM_FAC_TYPE_ID<br>CLM_SRVC_CLSFCTN_TYPE_CD	Days in which beneficiary is in a hospital-based hospice facility<br>CLM_FACT_TYPE_ID = 8 **AND**<br>CLM_SRVC_CLSFCTN_TYPE_CD = 2	CLM_FROM_DT to CLM_THRU_DT	Hospice
Visit	RBCS_Cat_Subcat	RBCS_Cat_Sub = EB, EE, EV, or EX;<br>Each day, count one visit per unique performing NPI (NCH: PRF_PHYSN_NPI, outpatient: AT_PHYSN_NPI) across NCH and outpatient files. If no NPI, do not count visit.<br>Includes evaluation and management visits for behavioral health services, ophthalmological services, office/outpatient services, and miscellaneous.	NCH: LINE_1ST_EXPNS_DT; if missing, CLM_THRU_DT<br>Outpatient: REV_CNTR_DT; if missing CLM_THRU_DT	Outpatient, NCH
Tests	RBCS_Cat	RBCS_Cat = T; includes anatomic pathology, cardiology, general laboratory, molecular testing,	Same as visit	Outpatient, NCH

© 2025 Gupta A et al. *JAMA Network Open*.

		neurologic, pulmonary function, and miscellaneous tests.		
Imaging	RBCS_Cat	RBCS_Cat = I; includes computerized tomography (CT), magnetic resonance (MR), nuclear, ultrasound, standard x-ray, and miscellaneous imaging.	Same as visit	Outpatient, NCH
Procedures	RBCS_Cat	RBCS_Cat = P or A; includes breast, cardiovascular, eye, digestive/gastrointestinal, hematology, musculoskeletal, other organ systems, skin, and vascular procedures, and anesthesia services.	Same as visit	Outpatient, NCH
Treatments	RBCS_Cat	RBCS_Cat=R or RBCS_Cat_SubCat = DG; includes chiropractic, dialysis, chemotherapy, injections and infusions (non-oncologic), radiation oncology, physical, occupational, and speech therapy, and miscellaneous treatments, and drugs administered through Durable Medical Equipment.	Same as visit	Outpatient, NCH
Chemotherapy[b]	RBCS_Cat_Subcat	RBCS_Cat_Subcat = RH	Same as visit; date within 180 days of diagnosis	Outpatient, NCH
Radiation therapy	RBCS_Cat_Subcat	RBCS_Cat_Subcat = RR	Same as visit; date within 180 days of diagnosis	Outpatient, NCH

[a] When identifying specific types of contact days, the following hierarchy was applied hospital/inpatient > emergency department > skilled nursing facility > inpatient hospice > any ambulatory care (e.g., if beneficiary was admitted to the hospital from the emergency department on a given day, it was only counted as an inpatient/hospital day).

[b] Treatment with radiation therapy and/or chemotherapy was measured in the 180 days following diagnosis with restructured BETOS Classification System taxonomy. The BETOS code for chemotherapy includes treatment with immunotherapy.

© 2025 Gupta A et al. *JAMA Network Open*.

eTable 2. Baseline Sociodemographic and Health-related Characteristics by Cancer Type (Secondary Cohort)

	Breast Cancer (n=11,246)[a]	Colorectal Cancer (n=25,425)[a]	Lung Cancer (n=110,593)[a]	Prostate Cancer (n=17,483)[a]
Age				
≤70	2,898 (25.77)	5,948 (23.39)	28,662 (25.92)	3,541 (20.25)
71-75	2,595 (23.07)	5,744 (22.59)	28,496 (25.77)	3,605 (20.62)
76-80	2,178 (19.37)	5,061 (19.91)	23,798 (21.52)	3,506 (20.05)
≥81	3,575 (31.79)	8,672 (34.11)	29,637 (26.80)	6,831 (39.07)
Sex				
Male	141 (1.25)	11,864 (46.66)	56,170 (50.79)	17,483 (100.00)
Female	11,105 (98.75)	13,561 (53.34)	54,423 (49.21)	0 (0.00)
Race/ethnicity				
Black	1,252 (11.13)	2,656 (10.45)	8,890 (8.04)	2,114 (12.09)
Hispanic	667 (5.93)	1,878 (7.39)	5,376 (4.86)	1,330 (7.61)
White	8,914 (79.26)	19,455 (76.52)	90,672 (81.99)	13,191 (75.45)
Other/unknown	413 (3.67)	1,436 (5.65)	5,655 (5.11)	848 (4.85)
Low-income subsidy[b]				
Full/partial	2,667 (23.72)	5,925 (23.30)	24,940 (22.55)	3,201 (18.31)
None	8,579 (76.28)	19,500 (76.70)	85,653 (77.45)	14,282 (81.69)
Urbanicity[c]				
Big metropolitan	6,542 (58.17)	14,371 (56.52)	59,494 (53.79)	9,585 (54.82)
Metropolitan	3,017 (26.83)	6,875 (27.04)	31,450 (28.44)	5,019 (28.71)
Urban	621 (5.52)	1,478 (5.81)	6,950 (6.28)	1,059 (6.06)
Less urban	897 (7.98)	2,252 (8.86)	10,539 (9.53)	1,503 (8.60)
Rural	169 (1.50)	449 (1.77)	2,160 (1.95)	317 (1.81)
Region[d]				
Northeast	4,001 (35.58)	8,979 (35.32)	35,756 (32.33)	5,709 (32.65)
Midwest	896 (7.97)	1,989 (7.82)	9,399 (8.50)	1,339 (7.66)
South	3,636 (32.33)	7,890 (31.03)	37,678 (34.07)	4,964 (28.39)
West	2,713 (24.12)	6,567 (25.83)	27,760 (25.10)	5,471 (31.29)
Comorbidities[e]				
0	5,699 (50.68)	10,819 (42.55)	34,239 (30.96)	8,226 (47.05)
1	2,281 (20.28)	5,499 (21.63)	26,122 (23.62)	3,176 (18.17)
≥2	3,266 (29.04)	9,107 (35.82)	50,232 (45.42)	6,081 (34.38)
Treated in 180 days after diagnosis[f]				
Yes	5,194 (46.19)	11,249 (44.24)	59,779 (54.05)	12,028 (68.80)
No	6,052 (53.81)	14,176 (55.76)	50,814 (45.95)	5,455 (31.20)
Year of Diagnosis[g]				
2008	972 (8.64)	2,561 (10.07)	11,322 (10.24)	1,230 (7.04)
2009	983 (8.74)	2,492 (9.80)	11,063 (10.00)	1,244 (7.12)
2010	975 (8.67)	2,448 (9.63)	10,695 (9.67)	1,286 (7.36)

© 2025 Gupta A et al. *JAMA Network Open*.

2011	981 (8.72)	2,381 (9.36)	10,342 (9.35)	1,322 (7.56)
2012	1,005 (8.94)	2,250 (8.85)	10,325 (9.34)	1,380 (7.89)
2013	1,001 (8.90)	2,220 (8.73)	9,938 (8.99)	1,511 (8.64)
2014	1,021 (9.08)	2,203 (8.66)	9,865 (8.92)	1,629 (9.32)
2015	1,010 (8.98)	2,228 (8.76)	9,703 (8.77)	1,767 (10.11)
2016	1,062 (9.44)	2,096 (8.24)	9,036 (8.17)	1,857 (10.62)
2017	1,018 (9.05)	2,071 (8.15)	8,761 (7.92)	1,987 (11.37)
2018	1,043 (9.27)	2,139 (8.41)	8,131 (7.35)	1,928 (11.03)
2019	175 (1.56)	336 (1.32)	1,412 (1.28)	342 (1.96)

[a] Cohorts included beneficiaries who were continuously enrolled in fee-for-service Medicare Parts A and B in the 12 months before and at least 1 month after diagnosis.
[b] Receipt of Medicare Part D low-income subsidies at diagnosis was an indicator of low income.
[c] Urbanicity was defined using rural-urban continuum codes known as Beale Codes. Beneficiaries missing urbanicity were categorized as Big Metropolitan (n=3 beneficiaries with lung cancer; n=1 beneficiary with prostate cancer).
[d] Surveillance, Epidemiology, and End Results registries were categorized into US census regions.
[e] Comorbidities were measured in the 12 months before and the month of diagnosis using the Klabunde modification of the Charlson score.
[f] Treatment with radiation therapy and/or chemotherapy was measured in the 180 days following diagnosis with restructured BETOS Classification System taxonomy. The BETOS code for chemotherapy includes treatment with immunotherapy.
[g] Cohort included beneficiaries diagnosed January 2008 to February 2019.

© 2025 Gupta A et al. *JAMA Network Open*.

eTable 3. Health Care Contact Days By Cancer Type (Secondary Cohort)[a]

	Breast Cancer (n=11,246)	Colorectal Cancer (n=25,425)	Lung Cancer (n=110,593)	Prostate Cancer (n=17,483)
	**Mean (SD)**	**Mean (SD)**	**Mean (SD)**	**Mean (SD)**
Total contact days[c]	40.84 (45.56)	43.28 (45.53)	36.53 (41.22)	39.02 (43.03)
Total institutional days[d]	16.52 (34.42)	19.85 (33.31)	15.74 (27.49)	15.40 (32.96)
Total ambulatory days[e]	24.32 (30.30)	23.43 (30.27)	20.78 (29.30)	23.62 (27.36)
Hospital/inpatient	6.33 (12.47)	9.40 (14.46)	7.86 (12.32)	5.98 (12.88)
Emergency department	0.74 (1.77)	0.79 (1.96)	0.83 (2.05)	0.99 (2.25)
Skilled nursing facility	7.88 (24.53)	7.49 (21.68)	4.95 (16.67)	7.25 (22.77)
Inpatient hospice	1.57 (15.61)	2.16 (16.58)	2.10 (14.82)	1.18 (12.92)
Visits	12.78 (13.25)	12.43 (14.99)	10.23 (13.27)	12.97 (11.85)
Tests	11.73 (13.23)	12.03 (14.76)	9.31 (12.75)	11.13 (10.99)
Imaging	4.03 (4.41)	2.99 (3.66)	3.37 (4.36)	3.54 (3.75)
Procedures	2.36 (5.17)	2.58 (5.20)	1.85 (3.92)	2.44 (4.07)
Treatment	14.00 (20.03)	12.55 (18.85)	12.64 (19.00)	12.41 (18.10)

Abbreviations: SD, standard deviation

[a] Health care contact days were measured until fee-for-service Medicare disenrollment, death, or study end (12 months after diagnosis). When identifying specific types of contact days, the following hierarchy was applied hospital/inpatient > emergency department > skilled nursing facility > inpatient hospice > any ambulatory care (e.g., if beneficiary was admitted to the hospital from the emergency department on a given day, it was only counted as an inpatient/hospital day).

[b] Mean percent was defined as the average of total days of each type of health care contact/follow-up time.

[c] Total institutional days are the sum of hospital/inpatient, emergency department, skilled nursing facility, and inpatient hospice days.

[d] Total ambulatory days are the sum of visits, tests, imaging, procedures, and treatment days.

[e] Total contact days are the sum of institutional and ambulatory days.

© 2025 Gupta A et al. *JAMA Network Open*.

eTable 4. Health Care Contact Days by Year of Diagnosis and Cancer Type (Primary Cohort)

		Breast Cancer (n=6,495)[a]	Colorectal Cancer (n=10,232)[a]	Lung Cancer (n=27,340)[a]	Prostate Cancer (n=11,739)[a]
		Mean (SD)	Mean (SD)	Mean (SD)	Mean (SD)
**2008**	Total[b]	47.39 (51.83)	61.69 (53.92)	59.47 (51.85)	37.85 (43.68)
	Institutional[c]	14.27 (39.17)	20.13 (40.51)	15.19 (34.57)	12.23 (32.27)
	Ambulatory[d]	33.12 (34.47)	41.56 (34.67)	44.28 (37.45)	25.63 (27.67)
**2009**	Total	47.52 (50.33)	64.17 (52.07)	60.87 (48.40)	36.44 (44.58)
	Institutional	13.53 (35.25)	21.28 (40.08)	13.86 (29.50)	10.84 (29.20)
	Ambulatory	34.00 (34.31)	42.89 (34.15)	47.00 (36.88)	25.61 (30.06)
**2010**	Total	46.05 (48.93)	61.76 (50.10)	60.41 (48.13)	38.12 (46.03)
	Institutional	13.26 (35.00)	19.94 (37.51)	14.15 (30.99)	12.18 (34.16)
	Ambulatory	32.79 (35.35)	41.82 (32.09)	46.26 (36.56)	25.94 (28.27)
**2011**	Total	48.26 (51.22)	59.35 (50.45)	59.06 (51.11)	36.79 (42.22)
	Institutional	14.07 (38.22)	17.19 (35.82)	13.79 (34.45)	11.19 (31.50)
	Ambulatory	34.19 (33.14)	42.16 (34.65)	45.27 (37.06)	25.60 (26.60)
**2012**	Total	46.26 (46.27)	59.31 (46.62)	58.53 (48.02)	38.62 (43.62)
	Institutional	12.58 (31.63)	17.84 (31.58)	12.54 (29.02)	11.25 (31.51)
	Ambulatory	33.68 (33.19)	41.47 (32.67)	45.99 (36.13)	27.37 (28.32)
**2013**	Total	46.18 (47.85)	58.85 (45.50)	58.43 (47.76)	37.38 (42.77)
	Institutional	12.94 (35.67)	15.74 (28.81)	13.34 (30.39)	10.10 (27.28)
	Ambulatory	33.23 (30.56)	43.11 (32.74)	45.09 (35.49)	27.28 (29.77)
**2014**	Total	44.88 (45.47)	62.29 (48.24)	57.65 (47.34)	38.66 (42.87)
	Institutional	11.58 (30.80)	18.69 (32.85)	13.28 (31.92)	10.49 (29.23)
	Ambulatory	33.29 (31.35)	43.60 (33.56)	44.37 (34.77)	28.17 (28.71)
**2015**	Total	43.92 (44.23)	58.61 (47.12)	58.46 (47.21)	39.28 (40.98)
	Institutional	10.48 (28.760	16.14 (33.58)	12.37 (29.72)	9.15 (24.86)
	Ambulatory	33.44 (31.99)	42.47 (32.14)	46.09 (35.33)	30.13 (30.42)
**2016**	Total	49.35 (45.59)	64.94 (46.32)	58.91 (42.64)	40.76 (41.59)
	Institutional	13.55 (31.82)	19.43 (35.51)	12.73 (28.44)	9.80 (28.05)
	Ambulatory	35.80 (33.72)	45.51 (31.02)	46.19 (32.07)	30.95 (28.83)
**2017**	Total	54.94 (43.05)	68.75 (39.77)	62.96 (39.41)	44.10 (40.18)
	Institutional	14.30 (32.05)	17.06 (27.66)	12.77 (25.44)	9.66 (24.76)
	Ambulatory	40.64 (30.63)	51.70 (29.32)	50.19 (29.85)	34.43 (29.14)
**2018**	Total	58.77 (51.16)	72.07 (44.02)	66.31 (42.89)	45.46 (40.47)
	Institutional	17.53 (43.83)	19.38 (36.41)	13.98 (31.33)	10.51 (27.81)
	Ambulatory	41.24 (30.87)	52.70 (28.94)	52.33 (30.93)	34.95 (27.52)
**2019**[e]	Total	61.55 (39.65)	73.56 (36.03)	67.43 (45.26)	47.09 (38.23)
	Institutional	15.34 (27.76)	17.94 (24.04)	15.07 (33.95)	11.42 (25.02)
	Ambulatory	46.21 (31.77)	55.62 (27.02)	52.37 (32.07)	35.66 (26.31)

Abbreviations: SD, standard deviation; IQR, interquartile range

[a] Cohorts included beneficiaries who were continuously enrolled in fee-for-service Medicare Parts A and B in the 12 before and after diagnosis.

[b] Total contact days are the sum of institutional and ambulatory days.

© 2025 Gupta A et al. *JAMA Network Open*.

[c] Total institutional days are the sum of hospital/inpatient, emergency department, skilled nursing facility, and inpatient hospice days.
[d] Total ambulatory days are the sum of visits, tests, imaging, procedures, and treatment days.
[e] Beneficiaries diagnosed from January 2019 to February 2019.

© 2025 Gupta A et al. *JAMA Network Open*.

eTable 5. Health Care Contact Days by Year of Diagnosis and Cancer Type (Secondary Cohort)

		**Breast Cancer (n=11,246)[a]**	**Colorectal Cancer (n=25,425)[a]**	**Lung Cancer (n=110,593)[a]**	**Prostate Cancer (n=17,483)[a]**
		**Mean (SD)**	**Mean (SD)**	**Mean (SD)**	**Mean (SD)**
**2008**	Total[b]	39.83 (47.76)	43.45 (48.83)	35.62 (42.45)	38.37 (45.91)
	Institutional[c]	16.96 (37.05)	21.59 (36.67)	16.78 (28.94)	18.21 (38.21)
	Ambulatory[d]	22.91 (31.02)	21.86 (30.62)	18.84 (28.93)	20.16 (25.76)
**2009**	Total	42.30 (49.42)	43.79 (47.37)	36.41 (42.22)	36.33 (44.00)
	Institutional	18.60 (38.62)	21.62 (36.29)	16.65 (28.32)	16.00 (33.00)
	Ambulatory	23.69 (30.99)	22.17 (30.13)	19.76 (29.67)	20.32 (27.29)
**2010**	Total	39.01 (46.16)	45.38 (48.31)	35.98 (42.34)	37.60 (45.79)
	Institutional	16.41 (34.84)	22.37 (37.17)	16.40 (29.12)	17.31 (37.41)
	Ambulatory	22.59 (31.62)	23.01 (30.00)	19.58 (29.30)	20.29 (25.99)
**2011**	Total	39.68 (46.40)	41.26 (45.33)	35.63 (42.68)	36.30 (42.71)
	Institutional	16.14 (35.12)	19.45 (33.01)	15.47 (28.73)	15.83 (34.34)
	Ambulatory	23.54 (30.20)	21.81 (30.44)	20.16 (29.73)	20.48 (24.90)
**2012**	Total	39.15 (44.98)	40.89 (44.94)	35.82 (41.06)	37.57 (44.42)
	Institutional	16.13 (32.67)	19.26 (32.77)	15.23 (26.62)	15.74 (35.10)
	Ambulatory	23.02 (30.35)	21.64 (29.38)	20.59 (29.47)	21.83 (26.45)
**2013**	Total	38.75 (45.03)	39.96 (43.23)	35.27 (41.48)	37.37 (44.61)
	Institutional	15.49 (34.64)	17.39 (29.51)	14.92 (27.28)	15.55 (34.27)
	Ambulatory	23.25 (28.41)	22.57 (30.08)	20.35 (29.43)	21.81 (27.62)
**2014**	Total	37.95 (43.31)	42.29 (45.21)	34.74 (40.23)	38.33 (43.18)
	Institutional	15.12 (32.32)	18.96 (31.89)	14.55 (26.40)	15.41 (32.78)
	Ambulatory	22.83 (28.76)	23.33 (30.38)	20.19 (28.69)	22.92 (27.25)
**2015**	Total	37.70 (42.96)	41.14 (44.56)	35.69 (41.03)	37.40 (41.97)
	Institutional	13.80 (30.11)	18.36 (32.43)	14.71 (26.59)	13.30 (29.67)
	Ambulatory	23.91 (30.19)	22.78 (29.60)	20.98 (29.55)	24.10 (29.14)
**2016**	Total	41.43 (44.03)	42.16 (44.17)	35.81 (38.62)	38.83 (40.92)
	Institutional	16.18 (31.40)	19.14 (31.87)	14.89 (25.33)	13.44 (29.08)
	Ambulatory	25.25 (30.92)	23.02 (29.67)	20.92 (28.21)	25.39 (27.91)
**2017**	Total	43.68 (41.98)	45.99 (42.80)	39.97 (38.88)	43.26 (41.81)
	Institutional	17.07 (30.88)	19.07 (29.75)	16.41 (25.97)	15.73 (31.99)
	Ambulatory	26.61 (29.56)	26.92 (30.47)	23.56 (28.68)	27.53 (28.10)
**2018**	Total	48.32 (48.71)	49.17 (44.09)	41.40 (40.71)	42.82 (40.46)
	Institutional	19.94 (49.67)	20.70 (33.16)	16.85 (27.75)	14.51 (30.37)
	Ambulatory	28.38 (30.28)	28.47 (30.91)	24.55 (29.88)	28.31 (27.25)
**2019[e]**	Total	46.65 (40.51)	48.76 (42.30)	43.00 (41.14)	46.77 (39.83)
	Institutional	15.49 (25.81)	17.58 (25.66)	17.38 (28.43)	16.90 (30.09)
	Ambulatory	31.17 (32.11)	31.18 (32.80)	25.63 (30.35)	29.87 (26.12)

Abbreviations: SD, standard deviation; IQR, interquartile range

[a] Cohorts included beneficiaries who were enrolled in fee-for-service Medicare Parts A and B in the 12 months before and at least 1 month after diagnosis.

[b] Total contact days are the sum of institutional and ambulatory days.

© 2025 Gupta A et al. *JAMA Network Open*.

[c] Total institutional days are the sum of hospital/inpatient, emergency department, skilled nursing facility, and inpatient hospice days.
[d] Total ambulatory days are the sum of visits, tests, imaging, procedures, and treatment days.
[e] Beneficiaries diagnosed from January 2019 to February 2019.

© 2025 Gupta A et al. *JAMA Network Open*.

eTable 6. Association of Sociodemographic and Health-related Characteristics with Health Care Contact Days (Primary Cohort)

	**Breast Cancer (n=6,495)**[a]		**Colorectal Cancer (n=10,232)**[a]		**Lung Cancer (n=27,340)**[a]		**Prostate Cancer (n=11,739)**[a]	
	**Mean (95% CI)**[b]	**IRR (95% CI)**[b]	**Mean (95% CI)**[b]	**IRR (95% CI)**[b]	**Mean (95% CI)**[b]	**IRR (95% CI)**[b]	**Mean (95% CI)**[b]	**IRR (95% CI)**[b]
Age								
≤70	49.44 (43.46-56.25)	Ref	49.93 (46.88-53.17)	Ref	42.79 (41.08-44.56)	Ref	30.85 (27.98-34.02)	Ref
71-75	49.11 (43.20-55.83)	0.99 (0.92-1.07)	49.41 (46.27-52.76)	0.99 (0.94-1.04)	42.79 (41.08-44.57)	1.00 (0.97-1.03)	30.55 (27.81-33.56)	0.99 (0.93-1.06)
76-80	46.77 (41.02-53.32)	0.95 (0.87-1.03)	50.23 (46.95-53.75)	1.01 (0.95-1.06)	43.76 (41.85-45.75)	1.02 (0.99-1.06)	30.56 (27.66-33.77)	0.99 (0.93-1.06)
≥81	45.50 (39.97-51.80)	0.92 (0.85-1.00)	57.49 (53.48-61.78)	1.15 (1.08-1.23)	46.47 (44.21-48.86)	1.09 (1.04-1.13)	30.38 (27.26-33.86)	0.98 (0.92-1.05)
Sex								
Male	46.72 (38.53-56.65)	Ref	49.87 (46.98-52.94)	Ref	42.20 (40.51-43.95)	Ref	-	-
Female	48.65 (44.90-52.72)	1.04 (0.87-1.24)	53.52 (50.36-56.88)	1.07 (1.03-1.12)	45.73 (43.97-47.56)	1.08 (1.06-1.11)	-	-
Race/ethnicity								
Black	55.12 (47.84-63.52)	1.47 (1.22-1.77)	58.90 (47.04-55.89)	1.43 (1.29-1.59)	48.09 (45.35-50.99)	1.30 (1.20-1.42)	34.78 (30.99-39.03)	1.35 (1.18-1.54)
Hispanic	37.57 (31.19-45.25)	Ref	41.17 (37.36-45.37)	Ref	36.92 (34.29-39.75)	Ref	25.79 (22.40-29.69)	Ref
White	53.22 (47.49-59.65)	1.42 (1.20-1.67)	57.29 (54.25-60.51)	1.39 (1.27-1.52)	47.84 (46.25-49.48)	1.30 (1.21-1.39)	34.26 (31.43-37.34)	1.33 (1.18-1.49)
Other/unknown	46.89 (39.42-55.76)	1.25 (1.01-1.54)	51.27 (47.04-55.89)	1.25 (1.11-1.39)	43.84 (41.30-46.53)	1.19 (1.09-1.30)	28.48 (25.32-32.04)	1.10 (0.96-1.27)
Low-income subsidy[c]								
Full/partial	50.00 (44.03-56.78)	1.10 (1.01-1.20)	56.17 (52.36-60.26)	1.18 (1.11-1.25)	46.25 (44.23-48.37)	1.11 (1.07-1.15)	34.61 (31.06-38.56)	1.28 (1.18-1.39)
None	45.47 (40.15-51.49)	Ref	47.52 (44.85-50.34)	Ref	41.72 (40.09-43.41)	Ref	27.03 (24.67-29.62)	Ref
Urbanicity[d]								
Big metropolitan	43.25	Ref	49.29	Ref	41.42	Ref	31.12	Ref

© 2025 Gupta A et al. *JAMA Network Open*.

	(39.02-47.93)		(47.15-51.53)		(40.13-42.77)		(29.37-32.97)	
Metropolitan	47.95 (42.58-53.98)	1.11 (1.03-1.19)	50.23 (47.62-52.98)	1.02 (0.97-1.07)	43.08 (41.46-44.77)	1.04 (1.01-1.07)	29.42 (27.55-31.43)	0.95 (0.90-0.99)
Urban	45.24 (37.21-55.02)	1.05 (0.87-1.26)	49.96 (45.94-54.33)	1.01 (0.94-1.10)	43.02 (40.49-45.69)	1.04 (0.98-1.10)	27.69 (24.96-30.73)	0.89 (0.81-0.98)
Less urban	48.33 (41.62-56.13)	1.12 (0.99-1.26)	52.24 (47.99-56.87)	1.06 (0.98-1.15)	44.55 (42.15-47.08)	1.08 (1.02-1.13)	28.38 (25.75-31.22)	0.91 (0.83-1.00)
Rural	54.35 (42.15-70.08)	1.26 (1.00-1.58)	56.96 (47.76-67.94)	1.16 (0.97-1.37)	47.82 (43.23-52.90)	1.15 (1.05-1.27)	37.19 (26.49-52.21)	1.20 (0.86-1.67)
Region[e]								
Northeast	55.98 (49.35-63.50)	1.93 (1.77-2.10)	60.45 (56.50-64.68)	1.84 (1.74-1.95)	49.73 (47.65-51.90)	1.68 (1.62-1.74)	36.39 (32.74-40.45)	1.77 (1.65-1.91)
Midwest	57.62 (49.95-66.47)	1.99 (1.78-2.22)	57.98 (53.38-62.98)	1.77 (1.63-1.91)	49.16 (46.44-52.04)	1.66 (1.57-1.75)	34.17 (30.64-38.11)	1.67 (1.51-1.84)
South	29.01 (25.42-33.11)	Ref	32.85 (30.91-34.92)	Ref	29.64 (28.47-30.86)	Ref	20.50 (18.61-22.59)	Ref
West	55.22 (48.82-62.46)	1.90 (1.74-2.08)	61.87 (57.98-66.02)	1.88 (1.77-2.00)	51.38 (49.24-53.62)	1.73 (1.67-1.80)	34.32 (30.92-38.10)	1.67 (1.55-1.80)
Comorbidities[f]								
0	32.81 (29.00-37.11)	Ref	37.01 (34.86-39.30)	Ref	30.16 (28.90-31.48)	Ref	21.03 (19.08-23.18)	Ref
1	50.64 (44.55-57.58)	1.54 (1.44-1.66)	54.78 (51.24-58.57)	1.48 (1.41-1.55)	47.94 (45.93-50.04)	1.59 (1.54-1.64)	31.27 (28.33-34.51)	1.49 (1.40-1.57)
≥2	65.24 (57.52-73.98)	1.99 (1.86-2.13)	68.01 (63.81-72.49)	1.84 (1.75-1.93)	58.61 (56.30-61.02)	1.94 (1.88-2.01)	43.51 (39.49-47.93)	2.07 (1.96-2.18)
Treated in 180 days after diagnosis[g]								
Yes	73.77 (65.51-83.08)	2.39 (2.24-2.55)	86.33 (81.87-91.03)	2.79 (2.64-2.96)	74.85 (72.31-77.48)	2.90 (2.79-3.02)	47.50 (43.80-51.50)	2.41 (2.22-2.62)
No	30.82 (27.16-34.97)	Ref	30.92 (28.74-33.26)	Ref	25.78 (24.54-27.08)	Ref	19.70 (17.51-22.15)	Ref
Year of Diagnosis[h]								
2008	44.90 (38.66-52.15)	Ref	52.01 (47.53-56.92)	Ref	42.90 (40.53-45.41)	Ref	29.57 (26.24-33.33)	Ref

© 2025 Gupta A et al. *JAMA Network Open*.

2009	47.08 (40.28-55.02)	1.05 (0.91-1.21)	54.27 (49.76-59.19)	1.04 (0.94-1.16)	42.86 (40.67-45.17)	1.00 (0.94-1.06)	28.56 (25.23-32.34)	0.97 (0.85-1.10)
2010	48.05 (41.25-55.97)	1.07 (0.93-1.23)	51.11 (46.93-55.67)	0.98 (0.89-1.09)	44.16 (41.69-46.77)	1.03 (0.97-1.10)	30.71 (26.46-35.64)	1.04 (0.91-1.19)
2011	46.09 (39.60-53.64)	1.03 (0.88-1.19)	47.37 (43.24-51.89)	0.91 (0.82-1.01)	40.68 (38.52-42.95)	0.95 (0.89-1.01)	26.79 (23.95-29.96)	0.91 (0.81-1.02)
2012	42.31 (36.56-48.96)	0.94 (0.82-1.08)	47.86 (44.09-51.95)	0.92 (0.83-1.02)	41.48 (39.27-43.80)	0.97 (0.91-1.03)	28.74 (25.52-32.37)	0.97 (0.86-1.10)
2013	41.74 (35.15-49.56)	0.93 (0.79-1.09)	46.02 (42.47-49.88)	0.88 (0.80-0.98)	41.16 (39.02-43.42)	0.96 (0.90-1.02)	26.56 (23.63-29.85)	0.90 (0.80-1.01)
2014	39.00 (33.64-45.21)	0.87 (0.76-0.99)	49.00 (45.07-53.27)	0.94 (0.85-1.04)	41.42 (39.08-43.90)	0.97 (0.91-1.03)	28.66 (25.60-32.09)	0.97 (0.86-1.09)
2015	40.60 (34.93-47.19)	0.90 (0.79-1.03)	45.81 (42.20-49.73)	0.88 (0.79-0.98)	40.84 (38.76-43.03)	0.95 (0.90-1.01)	28.35 (25.45-31.57)	0.96 (0.86-1.07)
2016	51.45 (44.64-59.29)	1.15 (1.01-1.31)	57.32 (52.81-62.21)	1.10 (0.99-1.22)	48.77 (46.09-51.62)	1.14 (1.07-1.21)	33.78 (29.98-38.07)	1.14 (1.02-1.28)
2017	55.90 (48.68-64.19)	1.25 (1.10-1.41)	58.93 (54.35-63.89)	1.13 (1.03-1.25)	46.61 (44.31-49.03)	1.09 (1.03-1.15)	35.28 (31.65-39.32)	1.19 (1.07-1.33)
2018	63.65 (54.47-74.37)	1.42 (1.23-1.64)	58.19 (53.76-62.99)	1.12 (1.02-1.23)	48.90 (46.49-51.44)	1.14 (1.08-1.21)	35.89 (32.36-39.80)	1.21 (1.09-1.35)
2019	57.62 (46.87-70.82)	1.28 (1.05-1.56)	54.45 (47.92-61.86)	1.05 (0.91-1.21)	48.67 (44.65-53.04)	1.13 (1.04-1.24)	36.37 (30.43-43.48)	1.23 (1.03-1.47)

Abbreviations: CI, confidence interval; IRR, incidence rate ratio

[a] Cohorts included beneficiaries who were continuously enrolled in fee-for-service Medicare Parts A and B in the 12 months before and after diagnosis.

[b] Predicted means and incidence rate ratios were estimated with negative binomial models that adjusted for sociodemographic and health-related factors.

[c] Receipt of Medicare Part D low-income subsidies at diagnosis was an indicator of low income.

[d] Urbanicity was defined using rural-urban continuum codes known as Beale Codes. Beneficiaries missing urbanicity were categorized as Big Metropolitan (n=3 beneficiaries with lung cancer; n=1 beneficiary with prostate cancer).

[e] Surveillance, Epidemiology, and End Results registries were categorized into US census regions.

[f] Comorbidities were measured in the 12 months before and the month of diagnosis using the Klabunde modification of the Charlson score.

[g] Treatment with radiation therapy and/or chemotherapy was measured in the 180 days following diagnosis with restructured BETOS Classification System taxonomy. The BETOS code for chemotherapy includes treatment with immunotherapy.

[h] Cohort included beneficiaries diagnosed January 2008 to February 2019.

© 2025 Gupta A et al. *JAMA Network Open.*

eTable 7. Association of Sociodemographic and Health-related Characteristics with Health Care Contact Days (Secondary Cohort)

	**Breast Cancer (n=11,246)[a]**		**Colorectal Cancer (n=25,425)[a]**		**Lung Cancer (n=110,593)[a]**		**Prostate Cancer (n=17,483)[a]**	
	**Mean (95% CI)[b]**	**IRR (95% CI)[b]**	**Mean (95% CI)[b]**	**IRR (95% CI)[b]**	**Mean (95% CI)[b]**	**IRR (95% CI)[b]**	**Mean (95% CI)[b]**	**IRR (95% CI)[b]**
Age								
≤70	44.58 (39.55-50.25)	Ref	42.61 (40.44-44.91)	Ref	29.99 (29.17-30.83)	Ref	30.99 (28.73-33.43)	Ref
71-75	43.54 (38.64-49.06)	0.98 (0.91-1.05)	41.48 (39.28-43.80)	0.97 (0.93-1.02)	29.38 (28.57-30.22)	0.98 (0.96-1.00)	32.11 (29.77-34.64)	1.04 (0.98-1.10)
76-80	40.69 (36.01-45.97)	0.91 (0.85-0.98)	40.81 (38.65-43.10)	0.96 (0.91-1.01)	29.15 (28.27-30.05)	0.97 (0.95-1.00)	30.29 (28.06-32.70)	0.98 (0.92-1.04)
≥81	37.44 (33.28-42.15)	0.84 (0.78-0.90)	40.37 (38.22-42.64)	0.95 (0.90-1.00)	28.47 (27.59-29.38)	0.95 (0.92-0.97)	30.01 (27.66-32.58)	0.97 (0.92-1.02)
Sex								
Male	41.55 (34.39-50.19)	Ref	40.02 (38.15-41.99)	Ref	27.33 (26.62-28.06)	Ref	-	-
Female	41.39 (38.58-44.39)	1.00 (0.83-1.19)	42.64 (40.61-44.76)	1.07 (1.03-1.10)	31.29 (30.47-23.13)	1.14 (1.12-1.17)	-	-
Race/ethnicity								
Black	47.16 (41.55-53.54)	1.45 (1.25-1.69)	49.13 (46.15-52.31)	1.29 (1.17-1.41)	34.32 (33.04-35.66)	1.49 (1.41-1.58)	36.86 (33.76-40.23)	1.48 (1.32-1.66)
Hispanic	32.47 (27.52-38.31)	Ref	31.94 (29.61-34.45)	Ref	23.01 (21.90-24.17)	Ref	24.90 (22.22-27.91)	Ref
White	45.02 (40.47-50.07)	1.39 (1.21-1.59)	45.15 (43.29-47.10)	1.41 (1.32-1.52)	29.83 (29.20-30.46)	1.30 (1.24-1.36)	33.86 (31.79-36.07)	1.36 (1.23-1.50)
Other/unknown	42.89 (36.66-50.19)	1.32 (1.11-1.58)	41.10 (38.13-44.31)	1.54 (1.41-1.67)	31.05 (29.70-32.45)	1.35 (1.27-1.44)	29.12 (26.31-32.23)	1.17 (1.03-1.32)
Low-income subsidy[c]								
Full/partial	42.45 (37.78-47.70)	1.05 (0.98-1.12)	44.38 (42.09-46.80)	1.15 (1.10-1.21)	30.43 (29.57-31.32)	1.08 (1.06-1.11)	33.90 (31.24-36.79)	1.21 (1.13-1.29)
None	40.50 (36.06-45.50)	Ref	38.45 (36.61-40.38)	Ref	28.10 (27.35-28.87)	Ref	28.06 (26.18-30.00)	Ref
Urbanicity[d]								
Big metropolitan	37.44	Ref	38.16	Ref	27.64	Ref	31.26	Ref

© 2025 Gupta A et al. *JAMA Network Open*.

	(33.87-41.39)		(36.91-39.44)		(27.07-28.21)		(29.78-32.81)	
Metropolitan	41.11 (36.82-45.90)	1.10 (1.03-1.17)	37.92 (36.34-39.57)	0.99 (0.96-1.03)	27.20 (26.52-27.90)	0.98 (0.96-1.01)	29.55 (27.99-31.19)	0.95 (0.90-0.99)
Urban	38.21 (32.57-44.82)	1.02 (0.89-1.17)	38.01 (35.17-41.09)	1.00 (0.92-1.08)	27.04 (25.94-28.19)	0.98 (0.94-1.02)	28.33 (25.91-30.98)	0.91 (0.83-0.98)
Less urban	44.01 (38.26-50.62)	1.18 (1.05-1.31)	43.66 (40.64-46.90)	1.14 (1.07-1.23)	29.49 (28.39-30.62)	1.07 (1.03-1.11)	28.99 (26.59-31.60)	0.93 (0.85-1.01)
Rural	47.38 (37.46-59.92)	1.27 (1.02-1.57)	50.09 (43.16-58.15)	1.31 (1.13-1.52)	35.68 (33.26-38.28)	1.29 (1.20-1.38)	36.80 (28.82-46.97)	1.18 (0.93-1.50)
Region[e]								
Northeast	50.16 (44.57-56.45)	2.01 (1.87-2.16)	50.32 (47.71-53.08)	2.02 (1.92-2.12)	34.56 (33.57-35.58)	1.90 (1.85-1.95)	37.47 (34.56-40.62)	1.88 (1.76-2.00)
Midwest	48.91 (42.98-55.66)	1.96 (1.78-2.15)	45.47 (42.35-48.82)	1.82 (1.70-1.96)	32.98 (31.76-34.24)	1.81 (1.75-1.88)	34.21 (31.25-37.45)	1.71 (1.57-1.87)
South	25.00 (22.13-28.23)	Ref	24.95 (23.67-26.29)	Ref	18.19 (17.69-18.71)	Ref	19.97 (18.54-21.51)	Ref
West	48.22 (42.89-54.22)	1.93 (1.78-2.09)	51.04 (48.49-53.67)	2.04 (1.94-2.15)	35.27 (34.29-36.28)	1.94 (1.88-2.00)	35.35 (32.72-38.20)	1.77 (1.66-1.89)
Comorbidities[f]								
0	28.90 (25.75-32.43)	Ref	29.75 (28.26-31.31)	Ref	19.40 (18.80-20.01)	Ref	21.13 (19.58-22.80)	Ref
1	46.21 (40.99-54.09)	1.60 (1.49-1.71)	45.52 (43.10-48.08)	1.53 (1.46-1.60)	33.87 (32.92-34.85)	1.75 (1.70-1.80)	32.74 (30.28-35.39)	1.55 (1.47-1.64)
≥2	53.40 (47.47-60.07)	1.85 (1.75-1.96)	52.06 (49.57-54.66)	1.75 (1.69-1.82)	38.06 (37.08-39.06)	1.96 (1.91-2.01)	42.41 (39.54-45.49)	2.01 (1.92-2.10)
Treated in 180 days after diagnosis[g]								
Yes	66.04 (59.08-73.82)	2.54 (2.41-2.67)	74.34 (71.11-77.71)	3.24 (3.13-3.35)	56.59 (55.23-57.98)	1.75 (3.67-3.82)	46.96 (44.11-49.99)	2.32 (2.18-2.46)
No	26.04 (23.16-29.27)	Ref	22.96 (21.80-24.18)	Ref	15.11 (14.68-15.56)	Ref	20.26 (18.60-22.07)	Ref
Year of Diagnosis[h]								
2008	39.54 (34.48-45.34)	Ref	42.57 (39.67-45.68)	Ref	28.01 (27.01-29.06)	Ref	31.31 (28.18-34.79)	Ref

© 2025 Gupta A et al. *JAMA Network Open*.

2009	43.96 (38.21-50.58)	1.11 (0.99-1.25)	43.79 (40.79-47.01)	1.03 (0.95-1.12)	28.86 (27.82-29.93)	1.03 (0.99-1.07)	28.84 (26.16-31.79)	0.92 (0.82-1.03)
2010	40.78 (35.49-46.85)	1.03 (0.91-1.16)	44.67 (41.62-47.95)	1.05 (0.97-1.14)	29.20 (28.07-30.37)	1.04 (1.00-1.09)	31.06 (27.70-34.83)	0.99 (0.88-1.12)
2011	39.32 (34.23-45.19)	0.99 (0.88-1.13)	38.03 (35.48-40.76)	0.89 (0.82-0.97)	27.04 (26.04-28.09)	0.97 (0.93-1.01)	27.28 (24.77-30.04)	0.87 (0.78-0.97)
2012	37.92 (33.10-43.44)	0.96 (0.85-1.08)	38.13 (35.58-40.86)	0.90 (0.83-0.97)	27.55 (26.53-28.61)	0.98 (0.94-1.02)	29.28 (26.63-32.19)	0.94 (0.84-1.05)
2013	37.18 (32.02-43.16)	0.94 (0.82-1.07)	36.25 (33.80-38.89)	0.85 (0.79-0.92)	27.16 (26.13-28.22)	0.97 (0.93-1.01)	28.34 (25.74-31.20)	0.91 (0.81-1.01)
2014	34.02 (29.72-38.94)	0.86 (0.77-0.96)	38.40 (35.83-41.14)	0.90 (0.83-0.98)	26.75 (25.75-27.80)	0.96 (0.92-1.00)	28.83 (26.40-31.48)	0.92 (0.83-1.02)
2015	37.38 (32.53-42.96)	0.95 (0.84-1.07)	37.31 (34.75-40.06)	0.88 (0.81-0.95)	27.25 (26.27-28.26)	0.97 (0.93-1.01)	27.46 (25.18-29.96)	0.88 (0.79-0.97)
2016	43.09 (37.83-49.08)	1.09 (0.97-1.22)	42.41 (39.60-45.42)	1.00 (0.92-1.08)	31.11 (29.90-32.37)	1.11 (1.06-1.16)	32.54 (29.64-35.73)	1.04 (0.94-1.15)
2017	46.86 (41.16-53.34)	1.19 (1.06-1.32)	44.90 (42.03-47.98)	1.05 (0.98-1.14)	32.87 (31.65-34.14)	1.17 (1.13-1.22)	35.42 (32.58-38.50)	1.13 (1.02-1.25)
2018	52.69 (45.83-60.57)	1.33 (1.18-1.51)	47.03 (43.99-50.29)	1.10 (1.02-1.20)	33.22 (32.03-34.46)	1.19 (1.14-1.24)	34.20 (31.49-37.13)	1.09 (0.99-1.20)
2019	48.63 (40.01-59.10)	1.23 (1.02-1.48)	43.97 (37.46-51.62)	1.03 (0.87-1.22)	33.07 (30.84-35.46)	1.18 (1.10-1.27)	37.40 (32.35-43.23)	1.19 (1.02-1.39)

Abbreviations: CI, confidence interval; IRR, incidence rate ratio

[a] Cohorts included beneficiaries who were continuously enrolled in fee-for-service Medicare Parts A and B in the 12 months before and at least 1 month after diagnosis.

[b] Predicted means and incident rate ratios were estimated with negative binomial models that adjusted for sociodemographic and health-related factors.

[c] Receipt of Medicare Part D low-income subsidies at diagnosis was an indicator of low income.

[d] Urbanicity was defined using rural-urban continuum codes known as Beale Codes. Beneficiaries missing urbanicity were categorized as Big Metropolitan (n=3 beneficiaries with lung cancer; n=1 beneficiary with prostate cancer).

[e] Surveillance, Epidemiology, and End Results registries were categorized into US census regions.

[f] Comorbidities were measured in the 12 months before and the month of diagnosis using the Klabunde modification of the Charlson score.

[g] Treatment with radiation therapy and/or chemotherapy was measured in the 180 days following diagnosis with restructured BETOS Classification System taxonomy. The BETOS code for chemotherapy includes treatment with immunotherapy.

[h] Cohort included beneficiaries diagnosed January 2008 to February 2019.

© 2025 Gupta A et al. *JAMA Network Open*.

eFigure 5. 12-Month Survival by Year of Diagnosis and Cancer Type (Secondary Cohort)

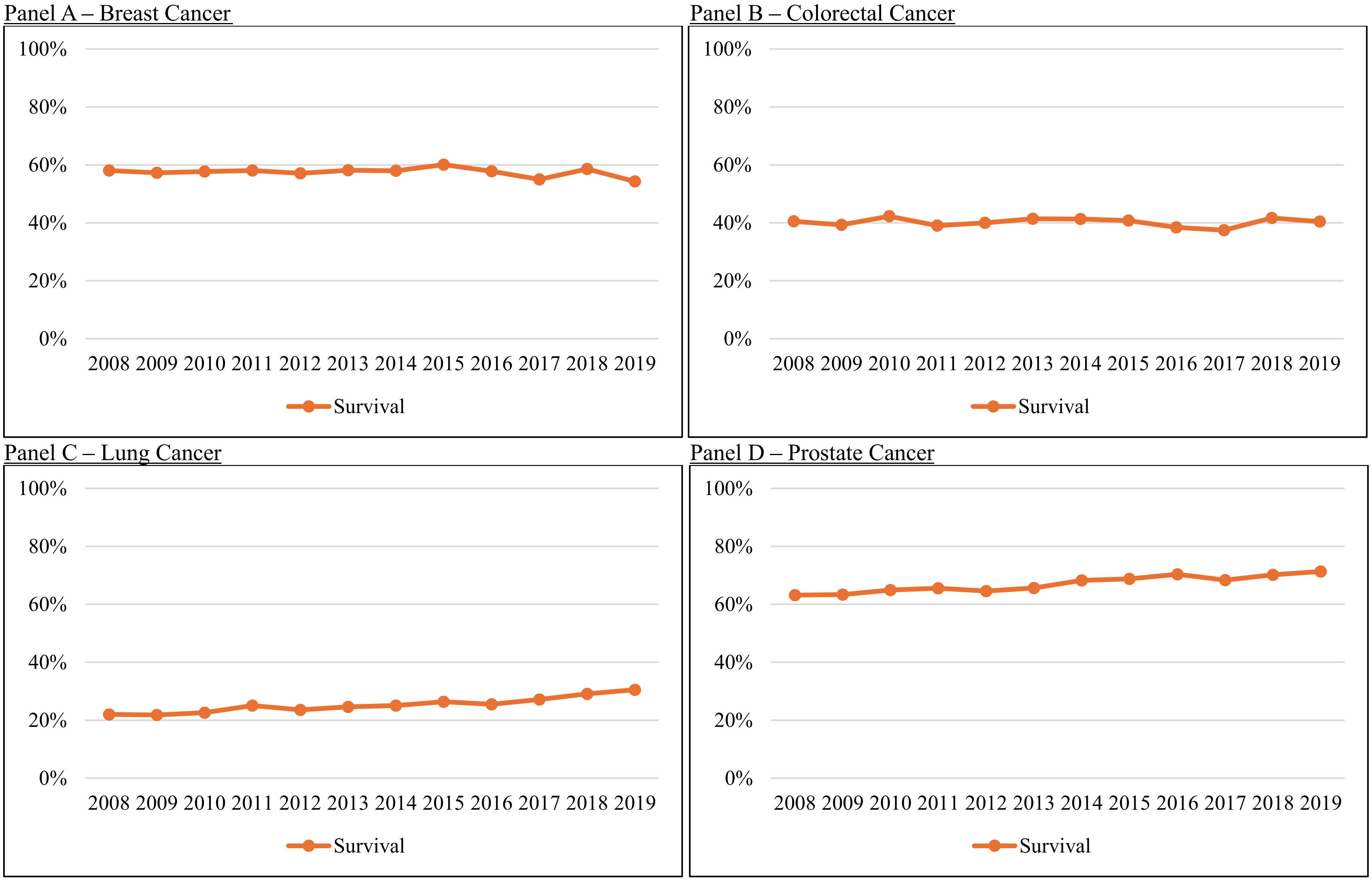

[a] Figure displays 12-month survival by year of diagnosis for beneficiaries who were continuously enrolled in Medicare Parts A and B in the 12 months before and at least 1 month after diagnosis.

© 2025 Gupta A et al. *JAMA Network Open*.
