# Supplementary material for: Health Care Contact Days in Older Adults With Metastatic Cancer
Source: JAMA Netw Open. 2025 Dec 9;8(12):e2547924. doi: 10.1001/jamanetworkopen.2025.47924 (PMC12690425; doi:10.1001/jamanetworkopen.2025.47924)
Supplement: Supplement 2. — Data Sharing Statement [file jamanetwopen-e2547924-s002.pdf]

Data Sharing Statement

Gupta. Health Care Contact Days in Older Adults With Metastatic Cancer. *JAMA Netw Open*. Published December 09, 2025. doi:10.1001/jamanetworkopen.2025.47924

Data
Data available: No

Additional Information
Explanation for why data not available: Medicare-SEER data use requires a DUA from Medicare
